# Site-Selective Incorporation of a Functional Group into Lys175 in the Vicinity of the Active Site of Chymotrypsin by Using Peptidyl α-Aminoalkylphosphonate Diphenyl Ester-Derivatives

**DOI:** 10.3390/molecules28073150

**Published:** 2023-03-31

**Authors:** Shin Ono, Masato Koga, Yuya Arimura, Takahiro Hatakeyama, Mai Kobayashi, Jun-ichi Sagara, Takahiko Nakai, Yoshikazu Horino, Hirofumi Kuroda, Hiroshi Oyama, Kazunari Arima

**Affiliations:** 1 Applied Chemistry, Kanazawa Institute of Technology, Hakusan 924-0838, Ishikawa, Japan; b1324923@planet.kanazawa-it.ac.jp (M.K.); b1640571@planet.kanazawa-it.ac.jp (Y.A.); b1366189@planet.kanazawa-it.ac.jp (T.H.); b1643910@planet.kanazawa-it.ac.jp (M.K.); 2 Applied Bioengineering, Kanazawa Institute of Technology, Hakusan 924-0838, Ishikawa, Japan; jun@neptune.kanazawa-it.ac.jp; 3 Graduate School of Science and Engineering, University of Toyama, Toyama 930-8555, Toyama, Japan; overcome.98per@gmail.com; 4 Department of Applied Chemistry and Bioscience, Chitose Institute of Science and Technology, Chitose 066-8655, Hokkaido, Japan; y-horino@photon.chitose.ac.jp; 5 Department of General Education, National Institute of Technology, Ishikawa College, Tsubata 929-0392, Ishikawa, Japan; kuroda@ishikawa-nct.ac.jp; 6 Faculty of Science and Engineering, Setsunan University, Hirakata 572-8508, Osaka, Japan; oyama@lif.setsunan.ac.jp; 7 Graduate School of Science and Engineering, Kagoshima University, Kagoshima 890-0065, Kagoshima, Japan

**Keywords:** chymotrypsin, dansyl group, diphenyl α-aminoalkylphosphonate, site-selective chemical modification

## Abstract

We previously reported that Lys175 in the region of the active site of chymotrypsin (Csin) could be site-selectively modified by using an *N*-hydroxy succinimide (NHS) ester of the peptidyl derivative containing 1-amino-2-ethylphenylphosphonate diphenyl ester [NHS-Suc-Ala-Ala-Phe^P^(OPh)_2_]. In this study, the Lys175-selective modification method was expanded to incorporate functional groups into Lys 175 in Csin. Two types of peptidyl phosphonate derivatives with the dansyl group (Dan) as a functional molecule, Dan-β-Ala-[Asp(NHS) or Glu(NHS)]-Ala-Ala-(*R*)-Phe^P^(OPh)_2_ (DanD and DanE, respectively), were synthesized, and their action was evaluated when modifying Lys175 in Csin. Ion-exchange chromatography (IEC), fluorescence spectroscopy, and LC-MS/MS were used to analyze the products from the reaction of Csin with DanD or DanE. By IEC and LC-MS/MS, the results showed that DanE reacted with Csin more effectively than DanD to produce the modified Csin (DanMCsin) bearing Dan at Lys175. DanMCsin exhibited an enzymatic activity corresponding to 1/120 of Csin against Suc-Ala-Ala-Phe-*p*NA. In addition, an effect of Lys175 modification on the access of the proteinaceous Bowman–Birk inhibitor to the active site of DanMCsin was investigated. In conclusion, by using a peptidyl derivative containing 1-amino-2-ethylphenylphosphonate diphenyl ester, we demonstrated that a functional group could be incorporated into Lys175 in Csin.

## 1. Introduction

Diphenyl α-aminoalkylphosphonates form a covalent bond with the hydroxy group of an active center Ser residue in serine proteases and then specifically inactivate serine proteases [1,2]. Because these diphenyl phosphonates are chemically stable in acidic conditions that are used for peptide synthesis, particularly Boc-chemistry, peptidyl derivatives bearing diphenyl α-aminoalkylphosphonates at their C-terminus have been synthesized to design potent irreversible inhibitors of serine proteases. The extended peptidyl sequences helped improve their specificity against the targeted enzymes [2,3,4,5]. Moreover, as reviewed by Sienczyk and Oleksyszyn, various potent and specific inhibitors for the urokinase-type plasminogen activator, human neutrophil elastase, mast cell chymase, dipeptidyl peptidase IV, herpesvirus protease, and others have been developed by introducing electron-withdrawing/donating substituents to the phenoxy rings of the phosphonates [6]. Diphenyl 1-amino-2-ethylphenylphosphonate, one of the diaryl α-aminoalkylphosphonate inhibitors, exhibits a selective inhibitory effect on chymotrypsin (Csin), and the peptidyl derivatives act as potent irreversible inhibitors.

Because diphenyl phosphonate derivatives show relatively high chemical stability in aqueous media, diaryl α-aminophosphonate derivatives have been employed as activity-based probes (ABPs) to characterize enzyme performance and detect localization in living cells. Targeted serine proteases could be visualized in living cells and tissues to evaluate their function in biological systems when fluorescence- and biotin-tags were added to the peptidyl phosphonate derivatives [6,7,8,9,10,11,12]. The combination of the peptidyl phosphonate derivatives and the detectable tags provides valuable imaging tools for investigating the function of serine proteases in live cells and tissues. Their reaction against serine proteases is potent and specific; however, because the reaction is irreversible, the labeled enzymes lose their enzymatic activity, limiting the use of the phosphonate derivatives as ABPs.

**Figure 1:**
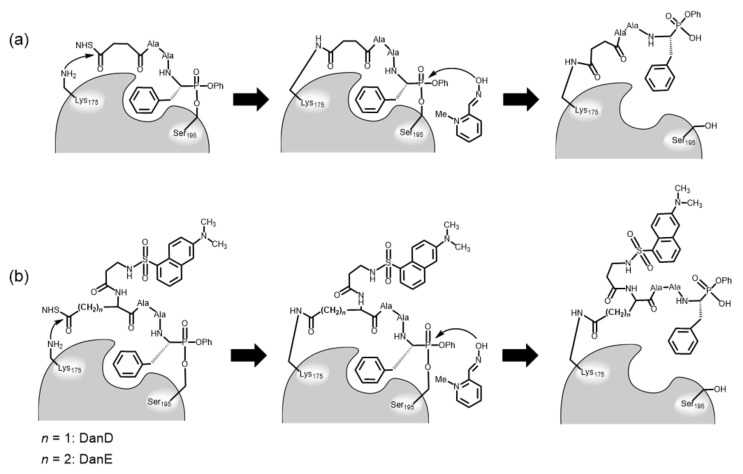
Site-selective chemical modification of Lys175 in Csin with the peptidyl phosphonate derivatives NHS-Suc-Ala-Ala-(*R*)-Phe^P^(OPh)_2_ (**a**), DanD and DanE (**b**).

We recently designed and synthesized an NHS-active ester of the peptidyl phosphonate derivative, [NHS-Suc-Ala-Ala-(*RS*)-Phe^P^(OPh)_2_], and demonstrated that the side chain of Lys175 in Csin could be site-selectively modified by this derivative (the modified Csin was referred to as MCsin) [13]. In this method, 2-pyridinaldoxime methiodide (2PAM) acted as a reactivator to break and replace the covalent bond between Ser195 and the phosphonate derivative, as previously described (Figure 1a). Furthermore, we used an optically active derivative [NHS-Suc-Ala-Ala-(*R*)-Phe^P^(OPh)_2_] to observe the effect of the unique structure on the Lys175 chemical modification. The study showed that the Lys175 chemical modification required the peptidyl phosphonate derivative containing a diphenyl phosphonate moiety with (*R*)-configuration [14]. These investigations imply that the peptidyl phosphonate derivatives are applicable for site-selective chemical modification of Csin and other similar enzymes.

Because site-selective chemical modification of proteins is an appealing and essential technique for studying protein processes in biological systems, several techniques for site-selective chemical modification of proteins have been developed to date [15,16,17,18,19,20,21,22,23,24,25]. Basle et al. reported that a trifunctional probe should be useful in preparing the desired protein bioconjugate, i.e., the combination of three distinct parts in a chemical modification molecule [17]. The probe molecules consist of a ligand or inhibitor part, a fluorescence or affinity tag part, and a reactive part for covalent bonding with proteins. In our site-selective chemical modification derivative, [NHS-Suc-Ala-Ala-(*R*)-Phe^P^(OPh)_2_], diphenyl phosphonates, and NHS-active ester moieties can function as a guide to the active site of Csin and a reactive part with Lys175, respectively. The fluorescence or affinity tag part is necessary to extend our peptidyl phosphonate derivative to such trifunctional probes for site-selective chemical modification.

In this study, two types of peptidyl phosphonate derivatives with a dansyl group (Dan) as a functional molecule were synthesized: Dan-β-Ala-[Asp(NHS) or Glu(NHS)]-Ala-Ala-(*R*)-Phe^P^(OPh)_2_ (DanD and DanE, respectively), and we investigated their action when modifying Lys175 in Csin. In these derivatives, Asp and Glu residues can provide two orthogonally reactive groups, namely, α-NH_2_ and side chain ω-COOH. We investigated whether the α-amino groups of Asp and Glu functioned as the third part that provided functional molecules (Figure 1b). Csin was treated with these derivatives, and the resulting products were analyzed to compare their suitability for the chemical modification of Lys175 in Csin. In addition, enzymatic activities of the finally obtained Csin-containing Dan group at Lys175 in the presence and absence of soybean Bowman–Birk inhibitor were examined to evaluate the influence of the chemical modification.

## 2. Results

### 2.1. Design of the Peptidyl Diphenylphosphonate Derivatives

We designed two types of peptidyl diphenylphosphonate derivatives, Dan-β-Ala-Xaa(NHS)-Ala-Ala-(*R*)-Phe^P^(OPh)_2_ (DanD and DanE with Asp and Glu, respectively), for the chemical modification of Lys175 in Csin based on the derivative NHS-Suc-Ala-Ala-(*R*)-Phe^P^(OPh)_2_ (Figure 1b) [13,14]. To introduce a dansyl group into Lys175, the w-carboxy groups of DanD and DanE were converted to NHS-active esters, and their a-amino groups were used for the introduction. Furthermore, b-Ala residue was selected to function as a spacer between the dansyl group and Asp or Glu residues. It was investigated which peptidyl phosphonate derivatives would be the most beneficial for introducing Dan into Lys175 in Csin.

**Figure 2:**
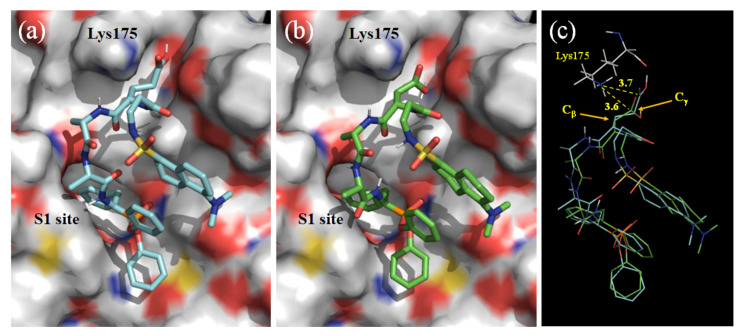
Estimated conformations of models DanE (**a**) and DanD (**b**) onto γ-Csin (PDB ID: 1gg6 [26,27]) with the lowest binding energies calculated via docking simulation by using AutoDock and superposition of models DanE and DanD (**c**). The distances between the ω-carboxy carbons of models DanD and DanE and the nitrogen of ε-amino group of Lys175 were estimated to be 3.6 and 3.7 Å, respectively.

Before the synthesis of these derivatives, DanD and DanE, a molecular simulation was performed to compare their applicability for the site-selective chemical modification by using the docking suite AutoDock, as previously reported [13,14]. Figure 2a,b show the estimated binding structures with the lowest binding mode energies −5.7 and −6.5 kcal/mol, for DanD (model DanD) and DanE (model DanE) without NHS esterification, respectively. These structures were similar, except for the position of the side chain carboxy groups, which differed slightly (Figure 2c). The binding mode energy of model DanE was estimated to be lower than that of model DanD, implying that DanE would modify Lys175 in Csin with a higher yield than that of DanD. Furthermore, in the case of DanE, the Glu side chain, which is more flexible due to the γ-carbon, may be advantageous in reacting with the side chain amino group of Lys175. The synthesis of the phosphonate derivatives is described in the Materials and Methods section.

### 2.2. Preparation of the Desired Csin Containing Dan Group at Lys175 (DanMCsin)

**Figure 3:**
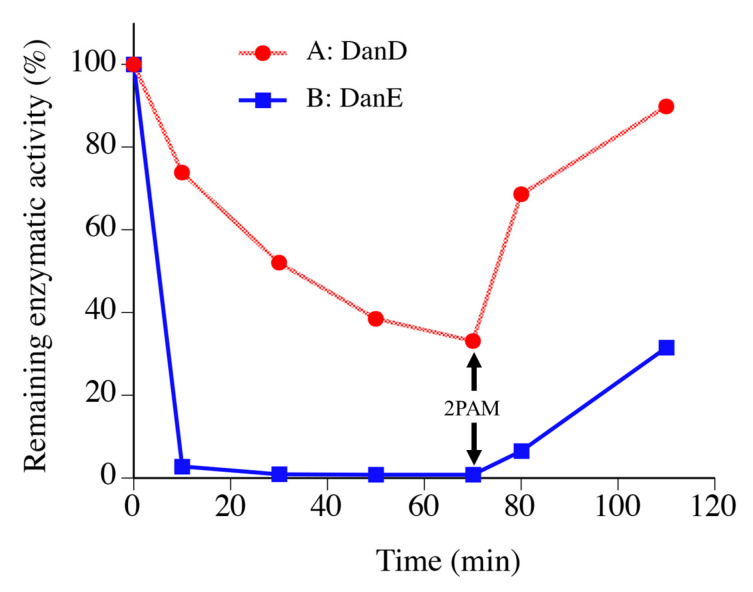
Changes in the remaining enzymatic activities during the modification process by using one equivalent of DanE (blue) or DanD (red) per Csin. The enzymatic activity was assayed by using Bz-L-Tyr-*p*NA as a substrate. The final substrate concentration was 0.2 mM. At 70 min, 2PAM was added to the reaction mixture.

To prepare chemically modified chymotrypsin, Csin was treated with one equivalent of DanD or DanE for 70 min. The remaining enzymatic activities for the reaction solutions were monitored by using Bz-L-Tyr-*p*NA as a substrate, according to a previous study [13]. Figure 3 shows the changes in the remaining enzymatic activities during the chemical modification. For the reaction solution with DanE, the remaining enzymatic activity rapidly dropped to 10% within the first 10 min, suggesting that more than 90% of the Csin was inactivated by the binding of DanE to the active site Ser195. To promote the selective chemical modification reaction at Lys175, the reaction mixture was left at room temperature for 70 min. After adding 2PAM to the reaction mixture at 70 min, the remaining enzymatic activity gradually increased to about 30% at 110 min.

In our previous work [13,14], we showed that Csin modified at Lys175 (MCsin) had undetectable activity against the substrate Bz-L-Tyr-*p*NA even after re-activation with 2PAM, whereas Csin that was modified only at Ser195 (and not Lys175) could be fully reactivated by 2PAM treatment. Our observation that the hydrolytic activity of DanE-treated Csin is undetectable during the modification procedure but can be partially recovered upon 2PAM treatment (~30%, Figure 3) is consistent with complete modification at Ser195 combined with incomplete modification at Lys175. In contrast, DanD showed a significantly different pattern; approximately 40% of the enzymatic activity persisted even at 70 min reaction time and more than 80% of the enzyme appeared to be reactivated after 2PAM treatment for 40 min (Figure 3). This is consistent with the presence of a higher proportion of Csin that is unmodified at both Ser195 and Lys175. The observed different reactivities of DanD and DanE toward Csin may be due to the different lengths and flexibilities of the Asp and Glu side chains.

**Figure 4:**
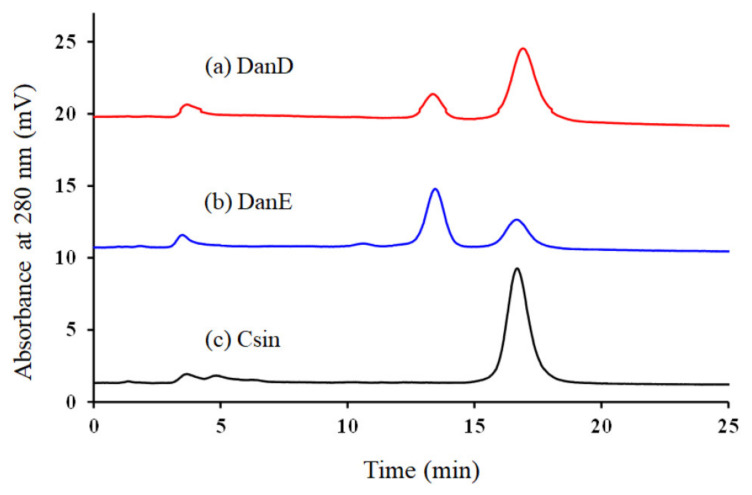
IEC profiles for the products after 2PAM treatment from the modification by using DanD (**a**, red), DanE (**b**, blue), and Csin (**c**, black). The separation was performed on 3 connected HiTrap^TM^ SP HP columns (7 mm I.D. × 25 mm) by using 50 mM sodium acetate buffer (pH 5.0) with a linear gradient of NaCl (0–0.5 M over 20 min). IEC analyses of the products obtained from DanD and DanE were performed 4 and 9 times, respectively.

To examine the modification reaction progress, the products from the reactions with DanD and DanE were analyzed on ion-exchange chromatography (IEC) after 2PAM removal via gel filtration. Figure 4 shows a comparison of typical chromatography profiles for DanD and DanE modification products. In the case of DanE, 2 distinct peaks appeared at 13.5 and 16.7 min (Figure 4b). The 13.5 min eluted component appeared to represent the desired product because the content eluted at 16.7 min was identical with native Csin (Figure 4c). The ratio of the areas for the 2 peaks, 13.5 min (MCsin) and 16.7 min (Csin), was 8:2, implying that the modification yield would be 80%. The modification reactions with DanE were performed 9 times, and the modification yields of the 13.5-eluted components ranged from 63 to 87%. Figure 4b shows the profile of DanE products giving the middle modification yield (80%) among those nine reactions. In the case of DanD, the modification reactions were repeated four times. The profile of DanD products is shown in Figure 4a when the highest yield (10%) of the 13.4 min eluted component was obtained out of 4 modification reactions. Though the 13.4 min eluted peak corresponding to modified Csin were also observed, the modification yields with DanD seemed to be lower than those with DanE.

**Figure 5:**
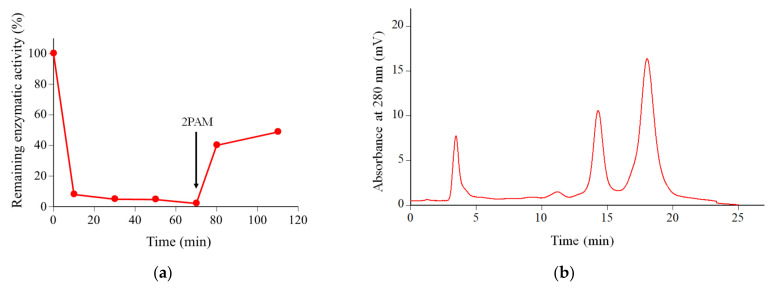
(**a**) Changes in the remaining enzymatic activity during the modification process by using two equivalents of DanD per Csin. The enzymatic activity was assayed by using Bz-Tyr-*p*NA dissolved in DMSO as a substrate. The final substrate concentration was 0.2 mM; (**b**) IEC profile for the product after 2PAM treatment from the modification by using 2 equivalents of DanD per Csin. The separation was performed on 3 connected HiTrap^TM^ SP HP columns (7 mm I.D. × 25 mm) by using 50 mM sodium acetate buffer (pH 5.0) with a linear gradient of NaCl (0–0.5 M over 20 min).

In addition, to confirm the low modification ability observed for DanD, we completely inactivated Csin by using two equivalents of DanD and examined the formation of the desired product. Figure 5a shows that most Csin was found to be inactive after 70 min, but more than 40% of the enzyme activity was restored after adding 2PAM. The IEC analysis for the resulting products showed that 40% of Csin (14.5 min) was observed to be modified, while the remaining 60% was observed to be unmodified (18.0 min), as shown in Figure 5b. These findings indicated that DanD provided a moderate modification yield at Lys175, even though most active sites of Csin were occupied by DanD.

All these results of the chemical modification experiments by using DanD and DanE demonstrate that DanE is more effective for selective modification of Lys175 in Csin than DanD. After the modification reaction of Csin by using DanE on a large scale, the component eluted at 13.5 min was collected from the IEC column as the desired modified Csin with the Dan group (referred to as DanMCsin) and used for further analyses.

### 2.3. Characterization of DanMCsin

#### 2.3.1. Fluorescent Property of DanMCsin

**Figure 6:**
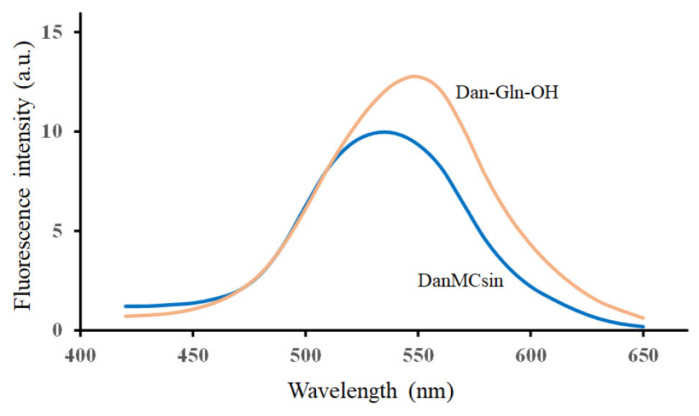
Fluorescence spectra of DanMCsin (blue) and Dan-Gln-OH (orange) in 40 mM ammonium acetate (pH 4.5) with an excitation wavelength at 336 nm.

To estimate the environment of the Dan group in DanMCsin, the fluorescence spectrum of DanMCsin was measured with an excitation wavelength at 336 nm (Figure 6) and compared with that of Dan-Gln-OH as a reference. In the fluorescence spectrum of DanMCsin in 40 mM ammonium acetate (pH 4.5), the emission maximum was observed at about 534 nm, while that of Dan-Gln-OH was observed at 550 nm. The blue shift of the emission maximum (16 nm) indicates that the Dan group in DanMCsin will be located under a relatively hydrophobic environment.

#### 2.3.2. Identification of the Site Modified by DanE

**Figure 7:**
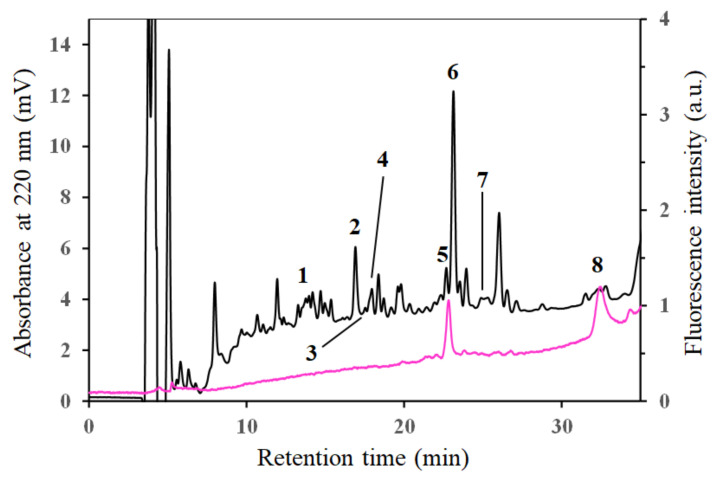
RP-HPLC analysis of the DanE modified Csin fragments after tryptic digestion. The profiles were monitored via UV detection (220 nm, black) and fluorescent detection (λ_ex_ 329 nm and λ_em_ 512 nm, magenta). The obtained fragments (peaks 1–8) were collected and subjected to ESI-TOFMS analysis.

**Figure 8:**
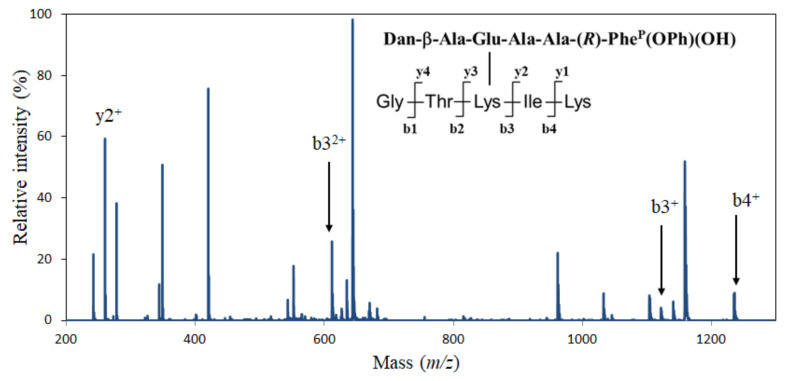
MS/MS analysis of the target ion (*m*/*z* 690.8). For identification of the modified chymotrypsins, the fragment molecular ion masses were analyzed with an electrospray ionization time-of-flight (ESI-TOF) mass spectrometer on a Hitachi NanoFrontier LC-MS system (Tokyo, Japan).

The synthesized DanMCsin was subjected to reduction, alkylation, tryptic digestion, and LC-MS/MS analysis to identify the modification site by DanE, as previously described [13,14]. Figure 7 shows the reversed-phase high-performance liquid chromatography (RP-HPLC) analysis profile for the digested fragments. The profile obtained via fluorescent detection (λ_ex_ 329 nm and λ_em_ 512 nm) implied that peak 5 at 23 min should be the target fragment containing the Dan group. The fractions (peaks 1–8) were collected and subjected to LC-MS/MS analysis. The targeted ion ([M + 2H]^2+^ *m/z* 690.8) for fragment GTK*IK (Figure S1), which contained the Dan group at the K* position, was observed among the components of peak 5 (Table S1). Moreover, the MS/MS analysis of the ion (*m*/*z* 690.8) indicated that the component was identical to the targeted molecule GTK*IK (Figure 8). These results demonstrate that Lys175 in Csin was selectively modified by DanE (Figure S2).

#### 2.3.3. Enzymatic Properties of DanMCsin

The hydrolytic activity of DanMCsin against Suc-Ala-Ala-Phe-*p*NA was measured to compare with that of the MCsin modified by NHS-Suc-Ala-Ala-(*RS*)-Phe^P^(OPh)_2_ [13]. Kinetic parameters (*K*_m_, *k*_cat_, and *k*_cat_/*K*_m_) for DanMCsin were estimated as presented in Table 1. The *K*m value of DanMCsin (0.95 mM) was similar to that of MCsin (0.85 mM), which shows that there is no significant influence of the Lys175 modification on the affinity of the modified Csin for a model substrate. The *k*cat value (1.5 s^−1^) increased about 6-fold (0.24 s^−1^ for MCsin). Comparing *k*_cat_/*K*_m_ values indicates that the enzymatic activity of DanMCsin will be 1/120th of that of Csin. In the case of MCsin, chemical modification at Lys175 also induced a significant decrease in enzymatic activity (lower than 1/300 compared with Csin). This suggests that further work to reduce the impact of the Lys175 modification on enzymatic activity will be required. Based on these similarities in Km combined with the decrease in *k*_cat_/*K*_m_ value, we speculate that the molecule bound to Lys175 of Csin does not reduce the enzymatic activity as a simple competitive inhibitor but, rather, affects the structure of the active site like an allosteric inhibitor, causing a large reduction in enzymatic activity. Such a hypothesis would be consistent with our computational analysis of the potential impact of Lys175 modification in the context of the complex of Csin with the Bowman–Birk Inhibitor (BBI).

**Figure 9:**
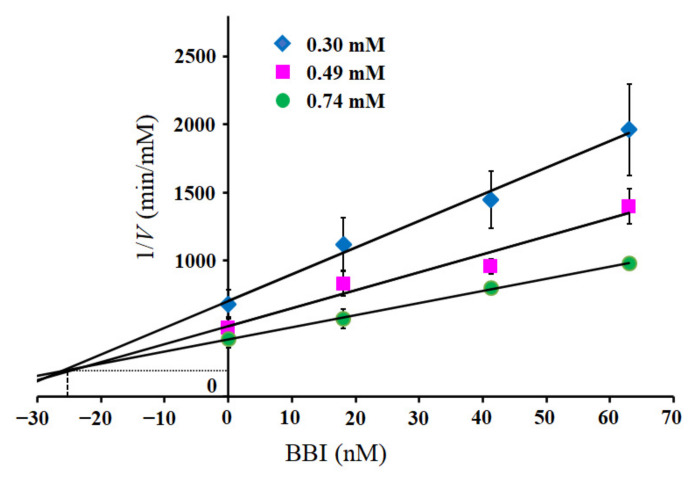
Dixon plots for the inhibition of DanMCsin with soybean Bowman–Birk inhibitor. Suc-Ala-Ala-Phe-*p*NA (0.30, 0.49, and 0.74 mM) was used as a substrate. Results are expressed as means ± SD of 3 experiments.

**Figure 10:**
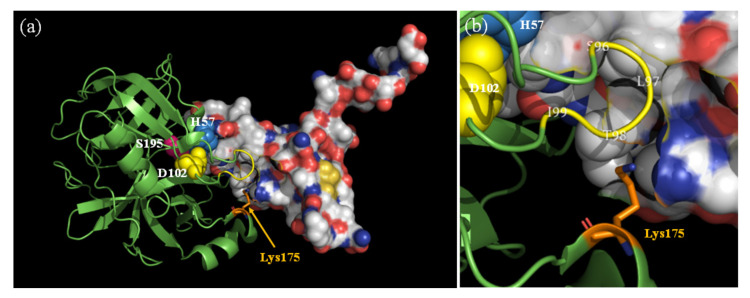
Crystal structure of Csin–BBI complex (PDB ID: 5J4Q [28,29]) by Johansson and Tornoee. (**a**) Structure of Csin (green) shown in solid ribbon format with catalytic triad (Ser195, magenta; HIs57, blue; Asp102, yellow) and Lys175 (stick, orange). Structure of BBI is shown in molecular surface and CPK format. (**b**) Diagram near Lys175. The side chain of Lys175 is in close proximity to the loop region (Ser96-Ile99, yellow).

The influence of the Dan group introduced into Lys175 in Csin on molecular recognition was evaluated by measuring the inhibitory activity of BBI against DanMCsin. The inhibitory constant, *K*_i_ value, of BBI for DanMCsin, was estimated to be 25 nM, whereas that of Csin was estimated to be 18 nM by using the Dixon plot (Figure 9). These data indicated that the Dan derivative bound to Lys175 did not disrupt the interaction between DanMCsin and BBI, although BBI is an inhibitory protein composed of 71 amino acids. This is supported by the structure of the BBI–Csin complex (Figure 10). Lys175 is located at the edge of the binding region between BBI and Csin but does not participate directly in the binding (Figure 10a). The side chain of Lys175 is close to the loop region (Ser96–Ile99) of Csin, but the ε-amino group is located on the surface (Figure 10b). Thus, DanE bound to Lys175 may be located outside of the BBI–Csin complex. Under the same conditions, we observed a variation in the intensity of emission maximum from the Dan group and sought to analyze it in detail. However, the intensity change was partial and insufficient to determine the mode of binding of BBI to DanMCsin. These results suggest that by employing our site-selective modification approach, functional groups can be introduced to Lys175 in Csin while retaining the intrinsic molecular recognition capacity.

## 3. Discussion

We showed that the Dan group could be incorporated into Lys175 in Csin by using the peptidyl α-aminoalkylphosphonate diphenyl ester derivative DanE, Dan-β-Ala-Glu(NHS)-Ala-Ala-(*R*)-Phe^P^(OPh)_2_, as a site-selective chemical modification molecule. The phosphonate moiety [(*R*)-Phe^P^(OPh)_2_] with (*R*)-configuration functions as a guide to lead and bind the entire derivative to the active site of Csin. The peptidyl spacer moiety (Ala-Ala) enhances the access of the NHS-active ester moiety in the Glu side chain to the ε-amino group of the side chain in Lys175. Consequently, an amide bond between the side chains of Glu and Lys175 residues is formed. Finally, by cleaving the P–O bond, 2PAM promoted the release of the phosphonate moiety from the active Ser195, and the site-selective chemical modification for Lys175 in Csin was completed. The modification yield (80%) was sufficient for the site-selective modification reaction in the case of DanE. However, it was unexpected that DanD would give the desired MCsin in a low yield (10%). The relatively higher reactivity observed for DanE over DanD was in good agreement with the simulations that used AutoDock. These results suggest the importance of the spatial position and/or flexibility of NHS-active ester in the modification molecule.

Site-selective chemical modification of proteins has been of great interest in biology, chemistry, and chemical biology within the last two decades [15,16,17,18,19,20,21,22,23,24,25]. Most research on site-selective chemical modification of proteins has concentrated on demonstrating protein localization and function of live cells. The use of ligand–protein interactions for selective chemical modification of proteins has been improving. For example, Hamachi et al. developed ligand-targeted chemical modification techniques in which a small ligand moiety guides the entire molecule to its binding site in the targeted protein, and a reactive moiety facilitates covalent bond formation at the selective site to introduce a reporter moiety [30,31,32,33,34,35]. In this study, peptidyl phosphonate derivatives were extended to trifunctional probes for site-selective chemical modification. However, our modification method cannot be applied to biological systems because 2PAM for the release of the phosphonate moiety bound to the active site is not applicable in cells. A minimum of 100 mM 2PAM is required to replace the phosphonate moiety that is bound to the active serine residue. It would be difficult to maintain such a high concentration of 2PAM in the cells because 2PAM is a polar compound that is not sufficiently permeable to the membrane. It is also necessary to consider the release of phenol from the diphenylphosphonate moiety as the modification reaction proceeds.

Our research develops artificial proteins with desired functions that are distinct from their intrinsic function by site-selectively integrating functional molecules. For this purpose, Csin is one of the most suitable proteins because of the following advantages. (1) The S1 site shows a unique ability to recognize aromatic rings such as phenyl and naphthyl groups. (2) Amide and ester bonds localized at an appropriate position in the active site can be hydrolyzed. (3) The three-dimensional structure has been analyzed and can be simulated. (4) The structure is relatively rigid and has a moderate tolerance to organic solvents that are used for syntheses. Our modification method that uses a combination of NHS ester of peptidyl diphenylphosphonate derivative and 2PAM rendered the site-selective incorporation of functional molecules into Lys175 in Csin achievable. Csin that contains a desired functional group at Lys175 can be prepared by replacing the Dan group in the derivative DanE with other functional molecules. From the inhibition study of DanMCsin by BBI, when a functional group is incorporated into Lys175, the resulting Csin will offer its active site for molecular recognition and hydrolytic action. Interestingly, Matsuo et al. reported the use of a mechanism-based inhibitor, L-phenylalanyl chloromethyl ketone, to introduce a Hoveyda–Grubbs catalyst into the cleft of Csin [36]. Intriguingly, the modified Csin demonstrated ring-closing metathesis catalytic activity in aqueous media, indicating the possibility of reconstructing Csin with a function that is different from the intrinsic one. Furthermore, several techniques for constructing artificial enzymes have been investigated thus far [37,38,39,40]. Moreover, this method can be applied for serine proteases other than Csin considering that the synthetic route for diaryl α-aminoalkylphosphonates corresponding to naturally occurring amino acids has been established [41].

## 4. Materials and Methods

### 4.1. General

Benzyl carbamate, triphenyl phosphite, and phenylacetaldehyde were purchased from Sigma-Aldrich (St. Louis, MO, USA). *N*^α^-Boc protected Ala, Lys, Glu, and Asp were obtained from Watanabe Chemical Industries Ltd. (Hiroshima, Japan). Coupling reagents, namely, 1-ethyl-3-(3-dimethylaminopropyl)carbodiimide monohydrochloride (EDC/HCl) and 1-hydroxybenzotriazole (HOBt), and *N*-hydroxysuccinimide (NHS) were purchased from the Peptide Institute Inc. (Osaka, Japan). Suc-Ala-Ala-Phe-*p*NA was synthesized from hydrochloric acid salt of Phe-*p*NA via a conventional method. *N*-tosyl-L-lysine chloromethyl ketone (TLCK)-treated Csin was purchased from Sigma-Aldrich (St. Louis, MO, USA). Thin layer chromatography (TLC) precoated plates were obtained from Merck (Tokyo, Japan). The solvent system that was used for TLC was chloroform-methanol-acetic acid (95:5:1). The concentration of Csin was determined by using a value A^1%^ (20.8) at 280 nm. Other chemicals were reagent grade.

### 4.2. Synthesis of the Chemical Modification Molecules DanD and DanE

DanD and DanE were synthesized via a conventional solution method described briefly as follows. A hydrobromide salt of racemic diphenyl (1-amino-2-phenylethy)phosphonate [(*RS*)-Phe^P^(OPh)_2_] was prepared according to the method proposed by Oleksyszyn [35] and coupled with Boc-Ala-OH to give the epimeric dipeptidyl derivative mixture. After deblocking the Boc-group, each epimeric derivative was separated on an RP-HPLC column by using a TFA-acetonitrile solvent system as previously reported [14]. The obtained dipeptidyl phosphonate (*R*)-epimer was used for further synthesis of the final products. Boc-Ala-OH and Boc-Glu(OtBu)-OH were successfully coupled with the dipeptidyl phosphonate (*R*)-epimer by using EDC-HOBt coupling and TFA deblocking reagents. Dan chloride was coupled with a methyl ester of β-alanine following alkaline hydrolysis, and the obtained Dan-β-Ala-OH was converted to an NHS active ester by using EDC. The TFA salt of H-Glu-Ala-Ala-(*R*)-Phe^P^(OPh)_2_ was reacted with Dan-β-Ala-NHS to give the corresponding Dansyl derivative. Then, the final product DanE was prepared via NHS active esterification with EDC and NHS. Esterification with NHS was achieved by using EDC-yielded DanE. The final derivative DanD was also synthesized with Boc-Asp(OtBu)-OH according to the same procedures described above. The purity of each compound synthesized was checked via TLC, RP-HPLC (Figure S3), ^1^H NMR (Figures S4 and S5), ^13^C NMR, and matrix-assisted laser desorption ionization time-of-flight (MALDI-TOF) mass spectrometry.

NMR spectra were recorded with a JNX-ECX500 spectrometer (JEOL, Tokyo, Japan). Chemical shifts (δ) were reported in ppm and coupling constants were reported in Hz with DMSO-*d_6_* referenced at 2.50 (^1^H) and 39.51 ppm (^13^C), respectively. Peak multiplicities were designated by the following abbreviations: s—singlet; d—doublet; t—triplet; q—quartet; m—multiplet; br—broad, and coupling constants are provided in (*J*) Hz. Molecular ion masses of the synthesized compounds were determined with a MALDI-TOF mass spectrometer on a Shimadzu AXIMA Performance (Kyoto, Japan) by using α-cyano-4-hydroxycinnamic acid as the matrix. Leu-enkephalin, angiotensin I, and neurotensin were used for calibration as the standards.

#### 4.2.1. Dan-β-Ala-Asp-Ala-Ala-(R)-Phe^P^(OPh)_2_

^1^H-NMR (500 MHz, DMSO-*d_6_*) δ 8.51 (d, *J* = 9.5 Hz, 1H), 8.45 (d, *J* = 8.5 Hz, 1H), 8.28 (d, *J* = 9.0 Hz, 1H), 8.25 (d, *J* = 7.5 Hz, 1H), 8.10 (d, *J* = 9.5 Hz, 1H), 7.93 (d, *J* = 6.0 Hz, 2H), 7.80 (d, *J* = 7.5 Hz, 1H), 7.63–7.57 (m, 2H), 7.41–7.36 (m, 4H), 7.29–7.14 (m, 12H), 4.78 (m, 1H), 4.48 (m, 1H), 4.28 (quint, *J* = 7.0 Hz, 1H), 4.18 (quint, *J* = 7.0 Hz, 1H), 3.28 (m, 1H) (partially overlap with water peak), 3.04 (q, *J* = 11.5 Hz, 1H), 2.96 (q, *J* = 11.5 Hz, 1H), 2.82 (s, 6H), 2.62 (dd, *J* = 5.0, 17.0 Hz, 1H), 2.40 (dd, *J* = 8.0, 16.5 Hz, 1H), 2.32–2.24 (m, 2H), 1.10 (d, *J* = 7.0 Hz, 3H), 1.04 (d, *J* = 7.0 Hz, 3H); ^13^C NMR (125 MHz, DMSO-*d_6_*) δ (appearance of extra carbon peaks is due to conformational isomers) 172.0, 171.97, 171.8, 171.3, 170.4, 169.9, 151.4, 149.98, 149.90, 149.88, 149.8, 136.9, 136.8, 135.7, 129.90, 129.87, 129.5, 129.1, 129.04, 129.0, 128.3, 128.2, 127.9, 126.5, 125.35, 125.3, 123.6, 120.61, 120.59, 120.43, 120.40, 119.0, 115.2, 49.4, 48.2, 48.0, 47.7, 46.5, 45.1, 36.0, 35.5, 34.2, 27.6, 18.2, 17.8.; MS (MALDI-TOF): 937.46 [M + Na^+^] (Calcd. 937.30); Anal. Calcd. for C_45_H_51_N_6_O_11_PS: C, 59.07; H, 5.62; N, 9.19%. Found: C, 59.06; H, 5.48; N, 9.12%.

#### 4.2.2. Dan-β-Ala-Glu-Ala-Ala-(R)-Phe^P^(OPh)_2_

^1^H-NMR (500 MHz, DMSO-*d_6_*) δ 12.1 (br s, 1H), 8.52 (d, *J* = 9.5 Hz, 1H), 8.45 (d, *J* = 8.0 Hz, 1H), 8.27 (d, *J* = 9.0 Hz, 1H), 8.10 (d, *J* = 7.5 Hz, 1H), 8.08 (d, *J* = 7.5 Hz, 1H), 8.00 (d, *J* = 7.0 Hz, 1H), 7.91 (t, *J* = 6.0 Hz, 1H), 7.80 (d, *J* = 8.0 Hz, 2H), 7.63–7.57 (m, 2H), 7.41–7.37 (m, 4H), 7.29–7.14 (m, 12H), 4.77 (m, 1H), 4.29 (quint, *J* = 7.0 Hz, 1H), 4.22–4.14 (m, 2H), 3.27 (m, 1H) (partially overlap with water peak), 3.03 (m, 1H), 2.98–2.92 (m, 2H), 2.82 (s, 6H), 2.30 (t, *J* = 7.5 Hz, 2H), 2.22 (t, *J* = 7.5 Hz, 2H), 1.82 (m, 1H), 1.67 (m, 1H), 1.11 (d, *J* = 7.0 Hz, 3H), 1.03 (d, *J* = 7.0 Hz, 3H); ^13^C NMR (125 MHz, DMSO-*d_6_*) δ (appearance of extra carbon peaks is due to conformational isomers) 174.0, 172.03, 172.00, 171.43, 170.96, 169.9, 151.4, 150.00, 149.92, 149.88, 149.8, 136.9, 136.8, 135.7, 129.9 (two carbons), 129.4, 129.07, 129.05, 129.02, 128.3, 128.2, 127.9, 126.5, 125.35, 125.29, 123.6, 120.61, 120.58, 120.41, 120.37, 119.0, 115.1, 51.9, 48.1, 47.9, 47.7, 46.5, 45.1, 35.4, 34.20, 34.18, 30.1, 27.3, 18.2, 17.8.; MS (MALDI-TOF): 951.33 [M + Na^+^] (Calcd. 951.31); Anal. Calcd. for C_46_H_53_N_6_O_11_PS: C, 59.47; H, 5.75; N, 9.05%. Found: C, 59.49; H, 5.73; N, 9.10%.

### 4.3. Mass Spectrometry

For identification of the modified chymotrypsins, the fragment molecular ion masses were analyzed with an electrospray ionization time-of-flight (ESI-TOF) mass spectrometer on a Hitachi NanoFrontier LC-MS system (Tokyo, Japan). A MonoCap C18 Fast-flow column (0.05 mm I.D. × 150 mm, GL Sciences, Tokyo, Japan) was used with a 0.1% formic acid–methanol solvent system at a flow rate of 200 nL/min.

### 4.4. Enzymatic Activity

The enzymatic activity was monitored by using a Hitachi U-1800 spectrophotometer (Tokyo, Japan) that was equipped with a thermostatted cell holder. The activity of Csin was assayed by using Suc-Ala-Ala-Phe-*p*NA dissolved in DMSO as a substrate. To prepare each buffer solution that contained a substrate at the desired concentration, 40 mM Tris-HCl containing 16 mM CaCl_2_ (pH 7.8), the substrate stock solution, and DMSO were mixed, and the mixture was incubated at 37 °C for 5 min. The enzyme solution was added to the mixture, and the production of *p*-nitroaniline was evaluated by monitoring the absorbance at 405 nm for 5 min (*p*-nitroaniline molar extinction coefficient 9920). The DMSO content was 10%. The final concentration of Suc-Ala-Ala-Phe-*p*NA was 0.30–2.0 mM. The concentrations of the phosphonate derivatives were determined based on the phenol released from them after alkaline hydrolysis. The concentration of phenol was quantified by using a calibration curve, which was generated for standard phenol via RP-HPLC.

### 4.5. Chemical Modification

To prepare chemically modified chymotrypsin, TLCK-treated bovine α-Csin (27 mM) was reacted with each derivative [DanD or DanE, 27 mM] for 70 min in Tris-buffer at 25 °C. Subsequently, 200 mM of 2PAM was added, and the mixture was incubated for 40 min at 4 °C. The final concentration of 2PAM used for reactivation was 100 mM. The remaining enzymatic activities for the reaction solutions were monitored by using Bz-L-Tyr-*p*NA as a substrate according to the previous study [13]. After incubation with 2PAM, some of the hydrolytic activity appeared to be restored. After gel filtration to remove the excess 2PAM, the fraction including native and modified Csin was collected and lyophilized. The components in the product obtained were separated via IEC, and a fraction that exhibited hydrolytic activity against Suc-Ala-Ala-Phe-*p*NA was collected and lyophilized. The modification reactions with DanD and DanE were performed 4 and 9 times, respectively. The product obtained was used for further analyses as the modified Csin. The total yields for the modified Csin were in the range of 20–30%.

### 4.6. Ion-Exchange and RP-HPLC Analyses

IEC analysis was performed on 3 connected HiTrap^TM^ SP HP columns (GE Healthcare, Uppsala, Sweden) (7 mm I.D. × 25 mm, Tosoh, Tokyo, Japan) by using 50 mM sodium acetate buffer (pH 5.0) with a linear gradient of NaCl (0–0.5 M). Each eluted component was separately collected and lyophilized. After gel filtration with 40 mM ammonium acetate (pH 4.5) and lyophilization, the purified product was dissolved in 50 mM sodium acetate (pH 5.0), and the solution was stored on ice to avoid autolysis. IEC analyses of the products obtained from DanD and DanE were also performed 4 and 9 times, respectively.

RP-HPLC analyses were performed by using a Shimadzu HPLC system, which comprised two LC-10ADvp pumps and an SPD 10Avp UV-Vis detector (Kyoto, Japan). For fluorescence monitoring, a Hitachi FL detector L-2485 (Tokyo Japan) was used. A TSKgel ODS-100V column (2.0 mm I.D. × 150 mm) and a COSMOSIL 5C18-AR-II column (20 mm I.D. × 250 mm, Nakarai Co., Osaka, Japan) were used for analytical and preparative RP-HPLC, respectively. A gradient comprising a TFA acetonitrile solvent system was used to evaluate the purity of the derivatives.

### 4.7. Inhibition by Bowman Birk Inhibitor

To determine the *K*_i_ value of BBI against DanMCsin, DanMCsin (10 µL, 12 µM) and BBI (10 µL, 2.9–10 µM) were mixed and incubated in a micro test tube at 25 °C for 10 min. An aliquot (10 µL) of the mixture was added to 790 µL of HEPES buffer containing Suc-Ala-Ala-Phe-*p*NA. The initial rates were obtained by monitoring the absorbance change at 405 nm for 5 min by using a Shimazu UV-Vis spectrometer. The measurements of the initial rates were conducted with three different final substrate concentrations (0.3, 0.49, and 0.74 mM), and subsequently, *K*_i_ value was evaluated from an analysis by using a Dixon plot. The *K*_i_ value of BBI was also obtained similarly.

### 4.8. Fluorescence Analysis

Fluorescence spectra of DanMCsin and Dan-Gln-OH were recorded with a Hitachi Fluorescence spectrophotometer F-2500 (Tokyo, Japan) at 25 °C. As a buffer, 40 mM ammonium acetate (pH 4.5) was used. The concentrations of DanMCsin and Dan-Gln-OH were 1.4 µM and 2.6 µM, respectively. Conditions: excitation wavelength: 326 nm; slit width: 2.5 nm (excitation), 5 nm (emission); response: 2.0 s.

## 5. Conclusions

A peptidyl diphenylphosphonate derivative bearing a fluorescent dansyl group, Dan-β-Ala-Glu(NHS)-Ala-Ala-(*R*)-Phe^P^(OPh)_2_(DanE), was synthesized and used for site-selective chemical modification of Lys175 in Csin. The product was purified on the IEC column, and the modification site was identified via LC-MS/MS analysis. DanE was observed to produce the desired product (DanMCsin) containing the dansyl group at Lys175 in 20–30% total yield. The enzymatic activity of DanMCsin was estimated to be about 1/120th of that of native Csin. The inhibitory activity of BBI against DanMCsin was similar to that against native Csin, indicating that the molecular recognition ability of the active site of DanMCsin was maintained after incorporating the dansyl group into Lys175. Therefore, our site-selective incorporation method that uses peptidyl diphenylphosphonate derivatives is expected to be used to construct artificial proteins by introducing functional molecules.

## Figures and Tables

**Table 1:** Kinetic parameters Csin and DanMCsin for Suc-Ala-Ala-Phe-*p*NA.

Enzyme	*K*_m_ (mM)	*k*_cat_ (s^−1^)	*k*_cat_/*K*_m_ (s^−1^M^−1^)
Csin ^1^	0.26 ± 0.02	49 ± 4.5	190,000
DanMCsin ^1^	0.95 ± 0.05	1.5 ± 0.32	1600

^1^ Kinetic parameters were measured in 50 mM HEPES containing 16 mM CaCl_2_ (pH 7.8). Results are given as means ± SD of 2–3 independent experiments.

## Data Availability

Not applicable.

## Supplementary Materials

The following supporting information can be downloaded at: https://www.mdpi.com/article/10.3390/molecules28073150/s1, Figure S1: ESI-TOFMS analysis for the tryptic fragment 5 from DanMCsin; Figure S2: Amino acid sequence of DanMCsin; Figure S3: Analytical RP-HPLC profiles of Dan-β-Ala-Asp-Ala-Ala-(R)-PheP(OPh)2 and Dan-β-Ala-Glu-Ala-Ala-(R)-PheP(OPh)2; Figure S4: 1H-NMR spectra of Dan-β-Ala-Asp-Ala-Ala-(R)-PheP(OPh)2; Figure S5: 1H-NMR spectra of Dan-β-Ala-Glu-Ala-Ala-(R)-PheP(OPh)2; Table S1: LC-MS/MS analysis of the fragments from DanMCsin after tryptic digestion.

## Sample Availability

Samples of the compounds (DanE and DanD) are available from the authors.
