# Supplementary material for: Site-Selective Incorporation of a Functional Group into Lys175 in the Vicinity of the Active Site of Chymotrypsin by Using Peptidyl α-Aminoalkylphosphonate Diphenyl Ester-Derivatives
Source: Molecules. 2023 Mar 31;28(7):3150. doi: 10.3390/molecules28073150 (PMC10096113; doi:10.3390/molecules28073150)
Supplement: Supplementary file 1 [file molecules-28-03150-s001.zip › molecules-2179711-supplementary.pdf]

Supplementary Materials

Site-Selective Incorporation of a Functional Group into Lys175 in the Vicinity of the Active Site of Chymotrypsin by Using Peptidyl α-Aminoalkylphosphonate Diphenyl Ester-Derivatives

Shin Ono[1,*], Masato Koga[1,†], Yuya Arimura[1,‡], Takahiro Hatakeyama[1,§] , Mai Kobayashi[1,||] , Jun-ichi Sagara[2], Takahiko Nakai[3,¶] , Yoshikazu Horino[4], Hirofumi Kuroda[5], Hiroshi Oyama[6], and Kazunari Arima[7,*]

Table of Contents

1. Identification of the modification site in DanMCsin

Figure S1. ESI-TOFMS analysis for the tryptic fragment 5 from DanMCsin.

Table S1. LC-MS/MS analysis of the fragments from DanMCsin after tryptic digestion.

Figure S2 . Amino acid sequence of DanMCsin.

2. Synthesis of Dan-β-Ala-Asp-Ala-Ala-(*R*)-Phe^{P}(OPh)$_2$ and Dan-β-Ala-Glu-Ala-Ala-(*R*)-Phe^{P}(OPh)$_2$.

Figure S3. Analytical RP-HPLC profiles of Dan-β-Ala-Asp-Ala-Ala-(*R*)-Phe^{P}(OPh)$_2$ and Dan-β-Ala-Glu-Ala-Ala-(*R*)-Phe^{P}(OPh)$_2$.

Figure S4. ^{1}H-NMR spectra of Dan-β-Ala-Asp-Ala-Ala-(*R*)-Phe^{P}(OPh)$_2$.

Figure S5. ^{1}H-NMR spectra of Dan-β-Ala-Glu-Ala-Ala-(*R*)-Phe^{P}(OPh)$_2$.

1. Identification of the modification site in DanMCsin

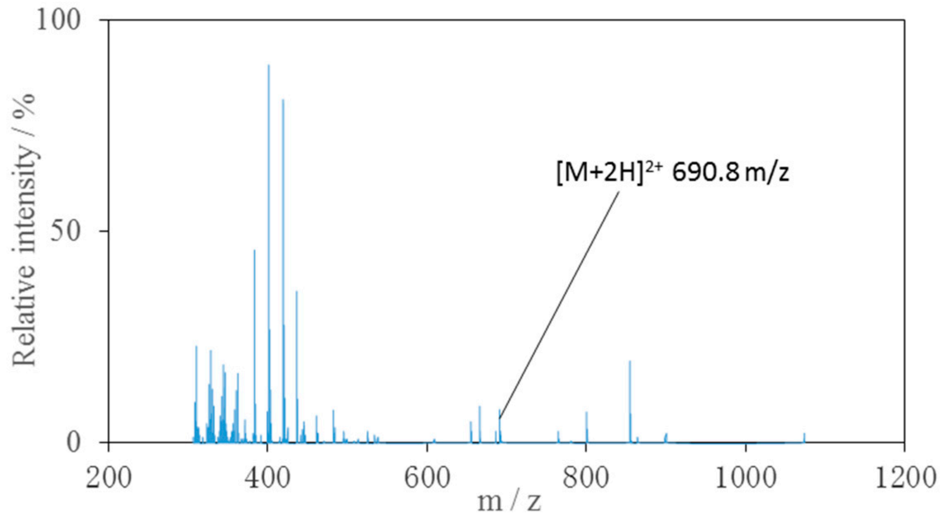

Figure S1 . ESI-TOFMS analysis for the tryptic fragment 5 from DanMCsin.

Table S1. LC-MS/MS analysis of the fragments from DanMCsin after tryptic digestion.

Fragment	Charge	Observed Ion (m/z)	Calc. Ion (m/z)	Deduced Sequence
1	2	770.87	1539.74	[16]IVNGEEAVPGSWPW[29]
	2	561.78	1121.57	[30]QVSLQDKTGF[39]
	2	869.96	1737.90	[147]TNANTPDRLQQASLPI[162]
	2	437.75	873.50	[208]TLVGIVSW[215]
2	2	412.70	823.44	[107]KLSTAASF[114]
3	3	541.33	1620.87	[94]YNSLTINNDITLLK[107]
4	2	799.99	1597.89	[229]ARVTALVNWVQQTL[242]
5	2	717.83	1433.65	[40]HFCGGSLINENW[51]
	2	690.8	1380.64	[173]GTK*IK[177]
	3	563.31	1686.86	[155]LQQASLPLLSNTNCK[169]
6	3	946.81	2837.30	[52]VVTAAHCGVTTSDVVVAGEFDQGSSSEK[79]
7	2	656.36	1310.69	[1]CGVPAIQPVLSGL[13]
8	3	931.13	2790.20	[115]SQTVSAVCLPSASDDFAAGTTCVTTGW[141]

The amino acid sequences determined by MS/MS analysis are shown. The observed mass and calculated mass of each peptide were identified from Mascot search results at www.matrixscience.com.

1 CGVPAIQPVL SGLSRIVNGE EAVPGSWPWQ 30
31 VSLQDKTGFH FCGGSLINEN WVVTAAHCGV 60
61 TTSDVVVAGE FDQGSSSEKI QKLKIAKVFK 90
91 NSKYNSLTIN NDITLLKLST AASFSQTVSA 120
121 VCLPSASDDF AAGTTCVTTG WGLTRYTNAN 150
151 TPDRLQQASL PLLSNTNCKK YWGTK*IKDAM 180
181 ICAGASGVSS CMGDSGGPLV CKKNGAWTLV 210
211 GIVSWGSSTC STSTPGVYAR VTALVNWVQQ 240
241 TLAAN

Figure S2 . Amino acid sequnece of DanMCsin.

The amino acid sequences denoted in red are the peptides determined by LC-MS/MS analysis. The fragment GTK*IK denoted in blue contains Lys175 at the K* position.

2. Synthesis of Dan-β-Ala-Asp-Ala-Ala-(R)-Phe^P(OPh)$_2$ and Dan-β-Ala-Glu-Ala-Ala-(R)-Phe^P(OPh)$_2$.

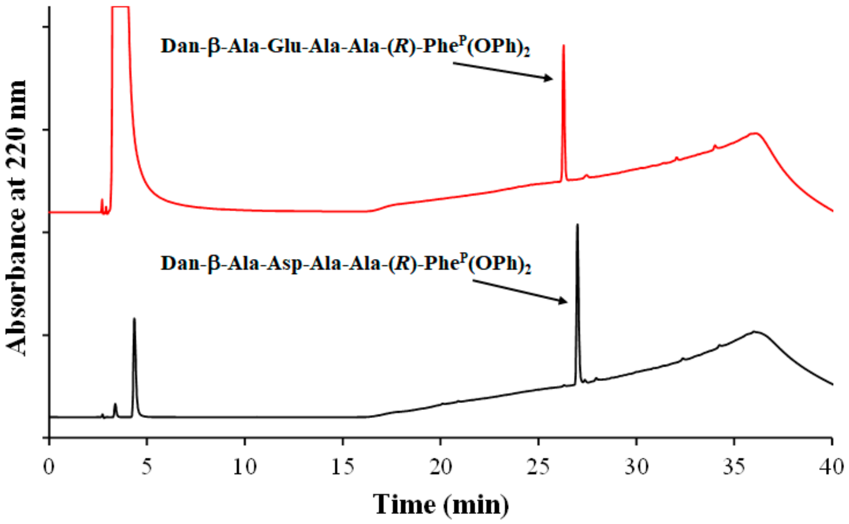

Figure S3. Analytical RP-HPLC profiles of Dan-β-Ala-Asp-Ala-Ala-(*R*)-Phe^P(OPh)$_2$ (black) and Dan-β-Ala-Glu-Ala-Ala-(*R*)-Phe^P(OPh)$_2$ (red).

A YMC-Triart C18 column (3.0 mm I.D. × 150 mm) with a gradient comprising a TFA-acetonitrile solvent system (10% acetonitrile/10 min and 10-30%/20 min). The eluted peaks (3-5 min) were from the solvent DMF (upper) and MeOH (lower).

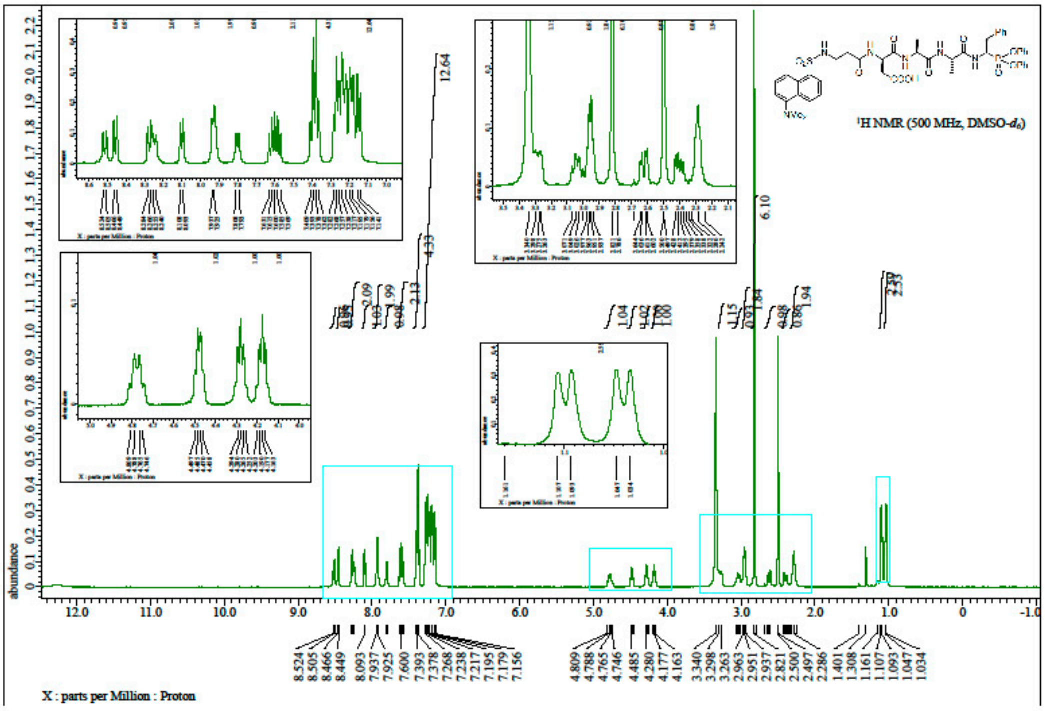

Figure S4. ¹H-NMR spectra of Dan-β-Ala-Asp-Ala-Ala-(*R*)-Phe[P](OPh)₂.

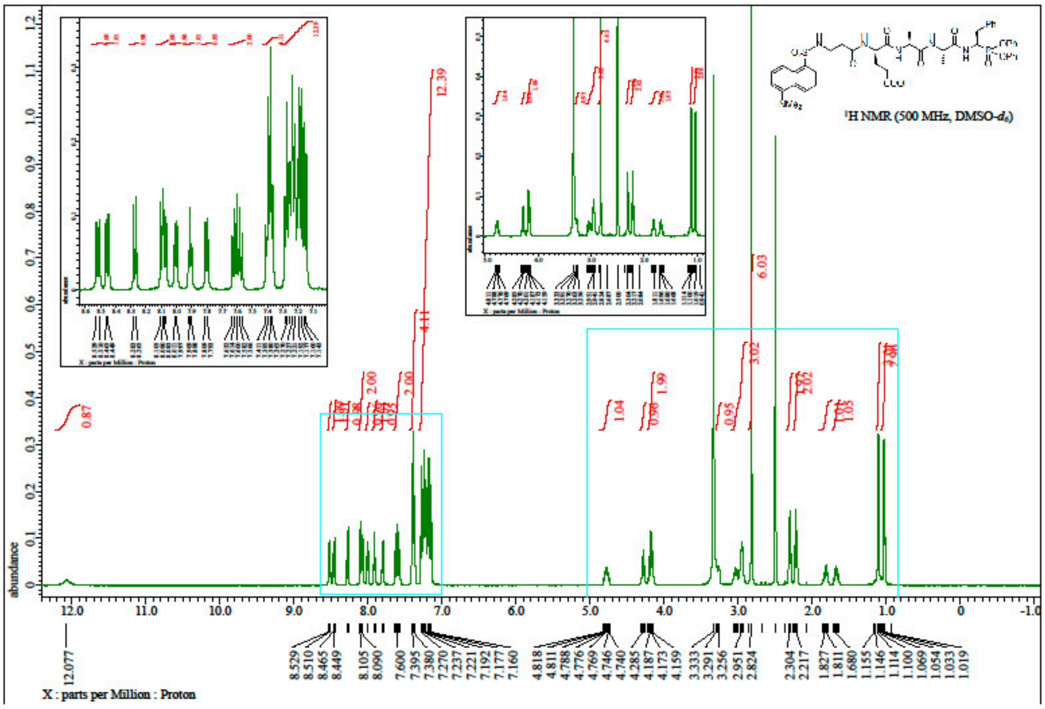

Figure S5. ¹H-NMR spectra of Dan-β-Ala-Glu-Ala-Ala-(*R*)-Phe[P](OPh)₂.
